# Bioactivity of Dietary Polyphenols: The Role in LDL-C Lowering

**DOI:** 10.3390/foods10112666

**Published:** 2021-11-02

**Authors:** Peng Sun, Liang Zhao, Nanhai Zhang, Jingxuan Zhou, Liebing Zhang, Wei Wu, Baoping Ji, Feng Zhou

**Affiliations:** 1 Beijing Key Laboratory of Functional Food from Plant Resources, College of Food Science and Nutritional Engineering, China Agricultural University, Beijing 100083, China; sunpeng666@cau.edu.cn (P.S.); nanhaizhang@cau.edu.cn (N.Z.); zjx888@cau.edu.cn (J.Z.); lbzhang@cau.edu.cn (L.Z.); jbp@cau.edu.cn (B.J.); 2 Beijing Advance Innovation Center for Food Nutrition and Human Health, Beijing Engineering and Technology Research Center of Food Additives, Beijing Technology and Business University, Beijing 100048, China; liangzhao@btbu.edu.cn; 3 College of Engineering, China Agricultural University, Beijing 100083, China; wuweiyin@cau.edu.cn

**Keywords:** cardiovascular diseases, LDL-C, metabolism pathways, dietary polyphenols

## Abstract

Cardiovascular diseases are the leading causes of the death around the world. An elevation of the low-density lipoprotein cholesterol (LDL-C) level is one of the most important risk factors for cardiovascular diseases. To achieve optimal plasma LDL-C levels, clinal therapies were investigated which targeted different metabolism pathways. However, some therapies also caused various adverse effects. Thus, there is a need for new treatment options and/or combination therapies to inhibit the LDL-C level. Dietary polyphenols have received much attention in the prevention of cardiovascular diseases due to their potential LDL-C lowering effects. However, the effectiveness and potential mechanisms of polyphenols in lowering LDL-C is not comprehensively summarized. This review focused on dietary polyphenols that could reduce LDL-C and their mechanisms of action. This review also discussed the limitations and suggestions regarding previous studies.

## 1. Introduction

Cardiovascular diseases (CVDs) have now become the leading cause of morbidity and mortality worldwide [1]. Some studies indicate that atherosclerosis is the principal pathogenic mechanism of CVDs [2,3]. An elevation of the low-density lipoprotein (LDL) cholesterol level in plasma is one of the most important risk factors for atherosclerosis [4]. To achieve optimal plasma LDL-C levels, huge amounts of therapies were investigated and implemented which targeted different metabolism pathways. For example, statins, the first-line drugs for lowering LDL-C levels, could inhibit the expression and activity of hepatic 3-hydroxy-3-methylglutaryl coenzyme A reductase (HMGR) and upregulate the expression and activity of the LDL receptor (LDLR) [5]. In clinical practice, however, about 50% of statins-treated patients do not achieve a desirable LDL-C level [6]. Thus, there is a need for new treatment options and/or combination therapies to suppress the plasma LDL-C level.

Studies have found that the intake of fruits and processed foods could directly reduce the LDL-C level, both of which were associated with their bioactive components like polyphenols [7,8,9]. Polyphenols refer to a large group of compounds containing aromatic ring(s) with one or more hydroxyl functional groups [10]. Based on their chemical structure, dietary polyphenols are mainly classified into phenolic acids, flavonoids, lignans, stilbenes, and phenolic polymers [11]. Flavonoids can be further divided into six subgroups: flavonols, flavones, isoflavones, flavanones, anthocyanidins, and flavanols [12]. In recent years, dietary polyphenols have received much attention in diseases prevention, such as type 2 diabetes, osteoarthritis, obesity, hyperlipidemia, hyperuricemia, etc. [13,14,15,16,17]. Our laboratory studies indicated that polyphenols could decrease plasma LDL-C levels in animal trials [18,19,20,21]. However, the effectiveness and potential mechanisms of polyphenols in lowering LDL-C is not comprehensively summarized. Therefore, in this review, we will discuss the effects and modes of action of dietary polyphenols on lowering LDL-C.

## 2. Metabolism Pathways for Lowering LDL-C

To achieve optimal plasma LDL-C levels, different metabolism pathways need to be considered. Currently, the metabolism pathways include the production and elimination of plasma LDL-C, the absorption of intestinal cholesterol, the production of very low-density lipoprotein (VLDL), the transfer of cholesterol esters through cholesterol esters transfer protein (CETP), the endocytosis of LDL via LDLR, and the formation and enterohepatic circulation of bile acids. Further details are mentioned below.

### 2.1. Intestinal Cholesterol Absorption

**Figure 1:**
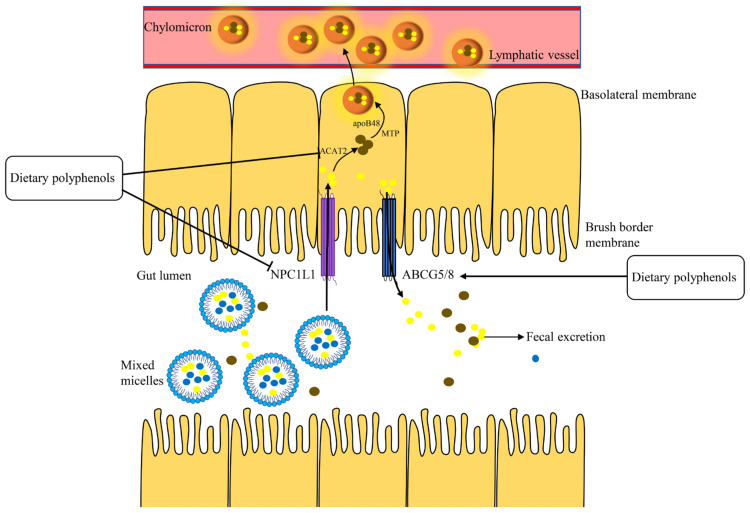
Intestinal cholesterol absorption. ACAT2, acyl-coenzyme A: cholesterol O-acyltransferase 2; MTP, microsomal triglyceride transfer protein; NPC1L1, NPC1-like transporter 1; ABCG5/8, adenosine triphosphate-binding cassette transporters G5 and G8.

There are two recognized sources of cholesterol in the body: biosynthesis and the intestinal absorption of dietary cholesterol [22,23]. Inhibiting cholesterol absorption had been shown to reduce the levels of cholesterol in the liver, thereby promoting the synthesis of LDLR (Section 2.4, below) and a subsequent reduction of plasma LDL-C levels [24]. Cholesterol absorption is a complex process which is regulated by a set of proteins: NPC1-like transporter 1 (NPC1L1), acyl-coenzyme A: cholesterol O-acyltransferase 2 (ACAT2), microsomal triglyceride transfer protein (MTP), and apolipoprotein B-48 (apoB48) (Figure 1) [25,26,27]. By contrast, some cholesterols are secreted back through adenosine triphosphate-binding cassette transporters G5 and G8 (ABCG5/8) [27]. Subsequently, the cholesterol is passed out from intestine [28].

### 2.2. VLDL Assembly and Secretion

**Figure 2:**
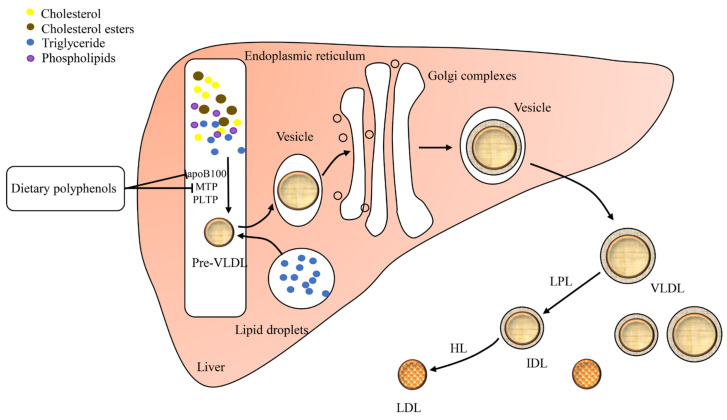
VLDL assembly and secretion. PLTP, phospholipid transfer protein; VLDL, very low-density lipoprotein; LPL, lipoprotein lipase; IDL, intermediate density lipoprotein; HL, hepatic lipase; LDL, low-density lipoprotein.

VLDL mainly delivers cholesterol and triglyceride from the liver to the bloodstream [29]. After processing in the bloodstream, VLDL is further translated into intermediate density lipoprotein (IDL) and then LDL particles by lipoprotein lipase (LPL) and hepatic lipase (HL) [30,31]. Thus, impairing liver VLDL production may reduce the rate of LDL production and the LDL-C level [32,33]. The assembly of VLDL involves a stepwise lipidation of apolipoprotein B100 (apoB100) in the liver (Figure 2) [34]. The first step of VLDL assembly is lipidation of apoB100 by MTP to generate pre-VLDL in the rough endoplasmic reticulum [35]. Moreover, the phospholipid transfer protein (PLTP) is also implicated in VLDL assembly and secretion through the blockade of apoB100 destruction [36,37]. The second step is the fusion of pre-VLDL with triglyceride-rich lipid droplets in the lumen of the smooth endoplasmic reticulum [38]. The nascent VLDL further moves from the endoplasmic reticulum to the Golgi and then transports from the Golgi to the plasma membrane [39].

### 2.3. CETP in LDL Metabolism

**Figure 3:**
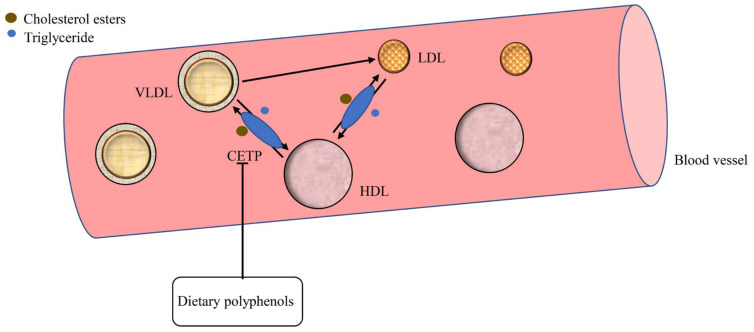
CETP-mediated LDL metabolism. CETP, cholesterol esters transfer protein; HDL, high-density lipoprotein.

Cholesterol esters transfer protein (CETP), a 476-residue hydrophobic glycoprotein, is synthesized mainly in the liver that engages the bidirectional transfer of cholesterol esters and triglycerides between lipoproteins [40]. The CETP promotes the transfer of cholesterol esters from high-density lipoprotein (HDL) to VLDL and LDL to exchange triglycerides (Figure 3). This produces triglycerides-enriched HDL and cholesterol-enriched LDL and VLDL are produced [41]. Inhibition of CETP activity can increase the concentration of HDL-C and lowers the concentration of LDL-C and VLDL-C in the plasma [42].

### 2.4. LDLR Mediates LDL Endocytosis

**Figure 4:**
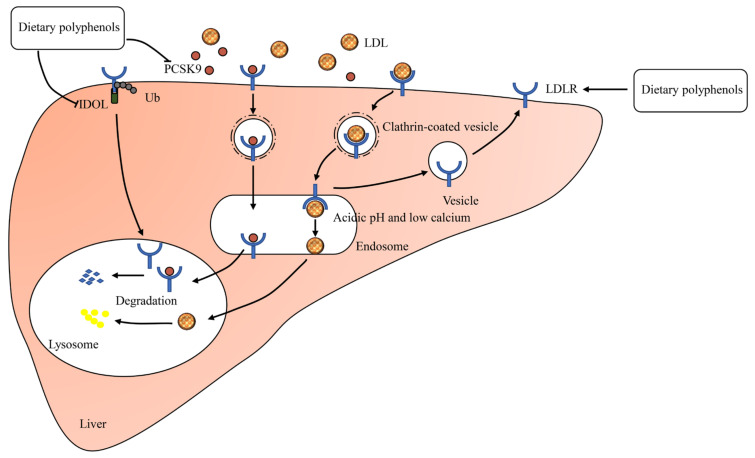
The regulatory mechanisms of LDL endocytosis. PCSK9, proprotein convertase subtilisin/kexin type 9; IDOL, inducible degrader of the LDLR; Ub, ubiquitin.

LDLR is a multidomain transmembrane protein, which is responsible for removing 70% of LDL-C from plasma [43]. Consequently, increased hepatic LDLR expression results in improved clearance of plasma LDL-C. The LDLR adhering to the liver surface binds LDL in the blood and LDL-LDLR complexes are internalized (Figure 4) [44]. Following internalization, the complexes are trafficked to sorting early endosomes where LDL release occurs [45]. Meanwhile, LDLR is either recycled back to the cell surface or transported to the lysosomes for degradation [46]. Degradation of LDLR is respectively regulated by two proteins: IDOL (inducible degrader of the LDLR) and PCSK9 (proprotein convertase subtilisin/kexin type 9) (Figure 4) [47,48].

### 2.5. Bile Acid Metabolism

**Figure 5:**
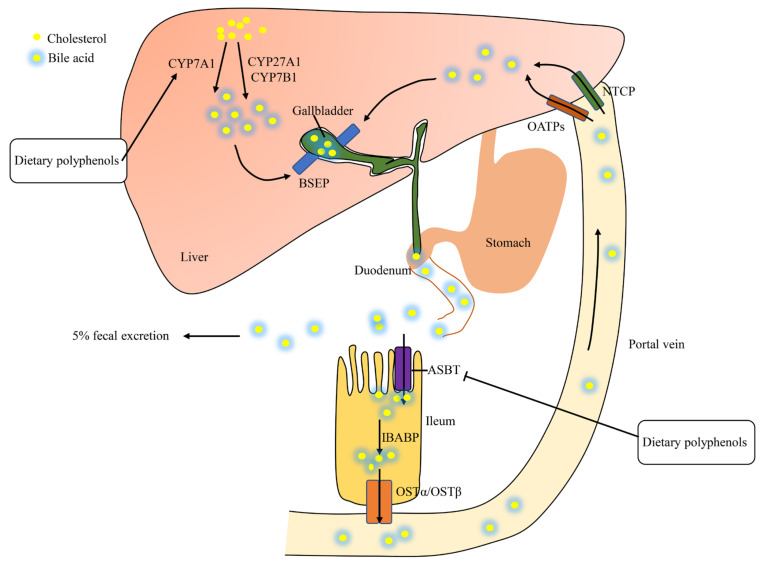
Enterohepatic circulation of bile acids. CYP7A1. cholesterol 7 α-hydroxylase; CYP27A1, sterol 27-hydroxylase; CYP7B1, oxysterol 7 α-hydroxylase; BSEP, bile salt export pump; OATPs, organic anion transporting polypeptides; NTCP, sodium-dependent taurocholate co-transporting peptide; ASBT, apical sodium-dependent bile acid transporter; IBABP, ileal bile acid binding protein; OSTα/OSTβ, organic solute transporters alpha and beta.

Bile acids, synthesized from cholesterol in hepatocytes, play a pivotal role in the absorption of dietary fats via the formation of micelles [49]. Hepatic cholesterol is transformed to bile acids via a variety of enzymes such as cholesterol 7 α-hydroxylase (CYP7A1), sterol 27-hydroxylase (CYP27A1), oxysterol 7 α-hydroxylase (CYP7B1), and so on (Figure 5) [50]. The newly synthesized bile acids are secreted to the canalicular space via the bile salt export pump (BSEP) to form bile [51]. Newly formed bile is stored in the gallbladder which is secreted into the duodenum in response to cholecystokinin [49]. A total of 95% of intestinal bile acids are reabsorbed via the apical sodium-dependent bile acid transporter (ASBT), the ileal bile acid binding protein (IBABP), and subsequently released into the portal vein through the organic solute transporters alpha and beta (OSTα/OSTβ) [52,53]. Hepatic uptake of bile acids is mediated by the sodium-dependent taurocholate co-transporting peptide (NTCP) and organic anion transporting polypeptides (OATPs) [52,54]. This recycling process is called enterohepatic circulation, which includes the liver, the biliary system, and the intestine [55]. Unabsorbed bile acids (about 5%) are excreted in feces, which stimulates the de novo synthesis of bile acids from cholesterol in liver [56]. Consequently, decreased hepatic cholesterol causes more LDL-C absorption from plasma to the liver [57,58].

## 3. Current Strategies for LDL-C Lowering

Up to now, numerous therapies have been developed to lower the LDL-C level. For example, statins are the first-line therapy in lowering LDL-C and can reduce the LDL-C level by about 60% [59]. Other strategies for LDL-C lowering include ezetimibe, bile acid sequestrants, anti-PCSK9 monoclonal antibodies (mAbs), bempedoic acid, squalene synthase inhibitors, mipomersen, lomitapide, ASBT inhibitors, and CETP inhibitors [59,60]. These strategies are mainly categorized into increasing the LDLR level, disturbing enterohepatic circulation, interfering VLDL assembly, and the CETP inhibitor [24,60,61,62,63,64,65,66,67]. Although these therapies are now reasonably effective, they have some shortcomings and adverse effects. For instance, anti-PCSK9 mAbs, mipomersen, and lomitapide are expensive, while statins and ezetimibe are relatively inexpensive as a series of generic drugs [59,68]. The long-term use of statins can also lead to muscle symptoms [69]. The details of current therapies are presented in Table 1.

## 4. Dietary Polyphenols: Potential LDL-C Lowering Agents

As previously mentioned, some therapies lowering the LDL-C level are effective but associated with adverse effects. Hence, alternative strategies are needed to reduce the high level of LDL-C effectively without any adverse effect. In recent years, dietary polyphenols have received much attention in diseases prevention due to their potential therapeutic effects. Dietary polyphenols are a class of phytochemicals containing phenol rings, mainly from fruits, vegetables, legumes, cereals, nuts, plant-derived beverages, and chocolate [13,74]. From the last few decades, numerous studies have suggested that dietary polyphenols have exhibited the potential of reducing plasma LDL-C levels [75,76,77]. In a recent animal study, polyphenols-rich *Perilla frutescens* leaf extracts (250 mg/kg body weight (bw)) were orally administrated to high-fat-diet-fed rats for 4 weeks. The results revealed that *Perilla frutescens* significantly reduced the plasma LDL-C level by 53% (*p <* 0.05) [78]. In an animal model study conducted by Li and colleagues revealed that the decrease of the plasma LDL-C content by 45% in the onion-fed group was associated with the inhibition of HMGR and the upregulation of LDLR expression, which was a plausible mechanism for its LDL-C lowering effect. Researchers concluded that this effect was due to the presence of quercetin and isoquercitrin in onion [79]. Polyphenols-rich grape extract capsules (300 mg/day) were orally given to obese individuals for a period of 12 weeks. The results predicted that grape extracts decreased the plasma LDL-C level from 131 ± 4.98 to 110.28 ± 5.8 mg/dl [80]. Luteolin, a bioactive compound present in chrysanthemum, was evaluated for its LDL-C lowering effect on hyperlipidemia rats. Luteolin (50 mg/kg bw) was orally given to male Sprague Dawley rats for 6 weeks. The results divulged that luteolin reduced the level of plasma LDL-C by 34% and increased the liver CYP7A1 activity in rats [81]. In this section, we will review the effect of diets rich in polyphenols on lowering LDL-C and focus on the underlying molecular mechanism of LDL-C lowering by dietary polyphenols based on in vitro or in vivo models.

### 4.1. Diets Rich in Polyphenols

Polyphenol supplementation in numerous studies contained mainly extracts (mulberry water extracts, aloe vera extracts, green tea extract, grape extract, etc.), juices (raspberry juice, apple juice, and chokeberry juice), wines, and an increased intake of polyphenol-rich foods (cacao, blueberry, and bilberry). In Table 2 and Table 3, we will focus on research published from 2011 to 2021 about the effect of polyphenol consumption on lowering LDL-C in animal and clinical studies, respectively.

### 4.2. Flavonoids

#### 4.2.1. Flavonols

Quercetin is a common plant flavonol which is widely present in vegetables, fruits, tea, and red wine [127]. In plants, partial quercetin has existed in the form quercetin-3-glucoside [128]. Quercetin exhibits favorable anti-atherosclerotic, anti-inflammatory, and antioxidant activities [129,130,131]. Interestingly, quercetin and its glycoside have attracted attention to the hypolipidemic effect [132,133,134]. In animal studies, treatment with quercetin and quercetin-3-glucoside significantly decreased the plasma LDL-C level in mice fed with a high-fat diet [133,134]. The quercetin group was administrated with quercetin in an aqueous solution through oral gavage with the dose of 12.5 mg/kg bw. Compared with the model group, the LDL-C level in quercetin group was reduced by 49% (*p <* 0.01). A total of 0.05% quercetin-3-glucoside supplementation significantly reduced the LDL-C level in comparison with the high-fat-fed control group. In a human study involving 24 subjects with mild hypercholesterolemia, the LDL-C level was greatly reduced by 9.2% after consuming 500 mL of quercetin-rich onion juice for 10 weeks (*p <* 0.01) [135]. The mechanisms by which quercetin and its glycoside inhibited the LDL-C level may involve several aspects. Firstly, quercetin reduced cholesterol absorption. The expression of NPC1L1, a critical protein in cholesterol absorption, was decreased in Caco-2 cells treated with 100 μM quercetin [136]. Secondly, both quercetin and its glycoside regulate LDLR expression. Moon demonstrated that 75 μM of quercetin upregulated the LDLR expression in HepG2 cells via the enhanced processing of the sterol regulatory element-binding protein 2 (SREBP2) following the sequential activation of c-Jun N-terminal kinases (JNK) and the extracellular signal-related kinase (ERK) signaling pathways [137]. In the Huh7 cells (human hepatocytes), treatment with 5 μM quercetin-3-glucoside accelerated the LDL uptake by increasing LDLR expression and attenuating PCSK9 secretion [138]. Similarly, Mbikay’s study showed that 0.05% quercetin-3-glucoside reduced PCSK9 secretion and increased the hepatic LDLR expression in mice fed with a high-cholesterol diet [134]. Thirdly, quercetin facilitates bile acid biosynthesis and excretion. In a Zhang’s study, it was suggested that a dietary supplementation of 0.4% quercetin increased the excretion of fecal bile acid and the level of CYP7A1, a critical enzyme promoting cholesterol-to-bile acid conversion in bile acid biosynthesis [139].

#### 4.2.2. Flavan-3-ols

Epigallocatechin-3-gallate (EGCG), which is an ester that forms through the reaction of epigallocatechin and gallic acid, is the major catechin in tea [140]. Substantial evidence suggests that EGCG elicit a wide range of properties, including antioxidant and anti-lipid deposition and anti-inflammation [141,142,143]. Extensive studies have shown that the consumption of EGCG exhibited an LDL-C reducing effect [144,145,146,147]. A systematic review found that EGCG could significantly decrease the plasma LDL-C level at doses between 107 and 856 mg/d [148,149,150]. Several mechanisms by which EGCG lowers the content of LDL-C involve cholesterol absorption, VLDL assembly, LDLR, and bile acid metabolism. Huang and colleagues [151] found that 0.32% EGCG reduced LDL-C by 28% in mice fed with a high-fat diet by increasing the fecal excretion of bile acids and cholesterol and decreasing cholesterol and bile acid reabsorption. Raederstorff and colleagues [152] found that EGCG affected intestinal cholesterol absorption by interfering with the micellar solubilization of cholesterol in a dose-dependent manner. Researchers [145] have pointed out that the enhanced biliary secretion of cholesterol was associated with the increased expression of ABCG5/ABCG8 and the decreased expression of ACAT2 by EGCG at a dose of 50 mg/kg bw. Moreover, evidence indicated that EGCG could suppress the LDL-C content by inducing an upregulation of LDLR [153]. A total of 25 μM EGCG could upregulate the level of LDLR through the ERK signaling pathways and downregulate the expression of PCSK9 in the HepG2 cell model [153]. In an animal study [147], rats fed with high-fat diet supplementation with 50 mg/kg bw of EGCG could weaken the concentration of circulation PCSK9 and nuclear factor-1α (HNF-1α) and the expression of PCSK9, as well as raise forkhead box class O (FoxO)3a and LDLR levels. Circulation PCSK9 could accelerate the degradation of LDLR, resulting in an elevated level of LDL-C in plasma [154]. HNF1α and FoXO3a are two transcription factors that regulate the expression of PCSK9 [147]. Thereby, EGCG acts on HNF1α and FoXO3a to reduce PCSK9 expression [147]. In a study by Kuhn, it was found that 1 μM EGCG directly inhibited ubiquitin/proteasome-mediated degradation of the active SREBP2, resulting in an elevated expression of LDLR [155].

#### 4.2.3. Anthocyanins and Proanthocyanidins

Anthocyanins are plentiful flavonoid pigments that occur in fruits, vegetables, and other plant-derived foods with a dark color, including blueberries, bilberries, blackcurrants, cranberries, cherries, and black rice [156]. Anthocyanins have been proven to possess antioxidant, anti-inflammatory, anti-atherosclerotic, and antiobesity properties [157,158,159,160]. Several epidemiologic studies have reported a marked decrease in LDL-C by the anthocyanin intervention [161,162,163,164,165]. According to previous research, daily supplementation of 90 mg, 320 mg, and 350 mg of purified anthocyanins or extracts could reduce the LDL-C level in hyperlipidemic patients by 8%, 13%, and 26.3%, respectively. No significant difference was observed in the plasma LDL-C level between patients with nonalcoholic fatty liver disease receiving anthocyanin or the placebo [166]. However, meta-analyses have found a statistically significant reduction of LDL-C in those individuals consuming anthocyanins [167,168]. Several mechanisms have been proposed to explain the effect of anthocyanins on lowering LDL-C. One possibility is that anthocyanins inhibit the activity of plasma CETP [162]. In a double-blind, randomized, and placebo-controlled trial, Qin and colleagues reported that the intake of 160 mg of anthocyanins twice daily significantly decreased the LDL-C concentration and the activity of CETP [162]. A second possibility is that anthocyanins could reduce the absorption of cholesterol [169]. In a hamster model of diet-induced dyslipidemia, anthocyanins at doses of 0.5% and 1% enhanced the fecal excretion of cholesterol accompanied with the downregulation on the gene expression of intestinal NPC1L1, ACAT-2, MTP, and ABCG8 [169]. Studies have consistently found that anthocyanins inhibited the formation of cholesterol micelles and increased the excretion of fecal sterols to lower the plasma LDL-C level [170,171,172]. The third possibility is that anthocyanin mediate bile acid metabolism [173]. In a study by Wang, it was found that dietary supplementation of 0.06% cyanidin-3-o-β-glucoside promoted fecal bile acid excretion and upregulated hepatic CYP7A1 expression compared with the control group [173].

Proanthocyanidins are known as condensed tannins, mainly present in fruits (berries, grapes, and apples), cereals, beans, and beverages (wine and tea) [174]. Proanthocyanidins were reported to consist of catechin, epicatechin, gallocatechin, and epigallocatechin-3-gallate, which divided into oligomers and polymers proanthocyanidins in terms of degree of polymerization [175]. Proanthocyanidins are believed to possess antiobesity, antioxidant, anti-inflammatory, anticancer, and hypolipidemic properties [176,177]. Previous studies showed that supplementation of proanthocyanidins could reduce both plasma TC and LDL-C levels [176,178]. The proanthocyanidins group was administrated with purified proanthocyanidins from lotus (*Nelumbo nucifera*, Gaertn) seed pot in an aqueous solution through an oral gavage with a dose of 25 mg/kg bw. Compared with the model group, the LDL-C level in the proanthocyanidins group was reduced by 48% (*p <* 0.05). The underlying mechanisms for lowering the plasma LDL-C level by proanthocyanidins are as follows. Firstly, proanthocyanidins were involved in the regulation of bile acid metabolism. For example, dietary supplementation of 1% grape seed proanthocyanidin increased the excretion of fecal bile acids and the upregulation of CYP7A1 in both the transcriptional and translational levels [179]. Similarly, supplementation with a high-cholesterol diet containing 1% cacao procyanidins could also enhance the fecal bile acid excretion in rats [180]. Heidker pointed out that grape seed procyanidin could not only enhance the expression of CYP7A1 but also decrease the absorption of intestinal bile acid by downregulating ASBT expression [181]. Secondly, the reduction of the plasma LDL-C level by proanthocyanidins (250 mg/kg bw) was associated with promoting the excretion of fecal cholesterol. The consumption of proanthocyanidins from cacao could inhibit the intestinal absorption of cholesterol through decreasing micellar cholesterol solubility and enhancing cholesterol excretion [180]. Thirdly, daily administration of 25 mg/kg bw of grape seed proanthocyanidins in rats had been shown to decrease the LDL-C level by 57%. The underlying mechanism involved disturbing VLDL assembling by repressing the expression of MTP and apoB [182].

#### 4.2.4. Curcumin

Curcumin, a hydrophobic polyphenol extracted from turmeric, has been widely used as a dietary spice by many cultures [183]. Curcumin was reported to exhibit broad spectral biological and pharmacologic activities including antioxidant, anti-inflammatory, antidiabetic, hypoglycemic, and antimicrobial capacities [184]. Moreover, curcumin was also shown to possess antihyperlipidemic activity [185,186]. Animal studies indicated that curcumin consumption could inhibit the LDL-C level in rats and hamsters fed with a high-fat diet [186,187,188,189]. After supplementation with 0.05% curcumin in male hamsters for 12 weeks, the LDL-C level was decreased by 34% compared to model group. Moreover, curcumin supplementation at a dose of 80 mg/kg bw could reduce the LDL-C level by 53% in obese rats [190]. Several human studies showed that curcumin could significantly decrease the plasma level of LDL-C in healthy middle-aged people and in patients with polycystic ovary syndrome, nonalcoholic fatty liver disease, mild chronic obstructive pulmonary disease, or type 2 diabetes [185,191,192,193,194]. Daily 180 mg curcumin supplementation in subjects with mild COPD was effective in diminishing the LDL-C level by approximately 11%. The LDL-C level in patients with nonalcoholic fatty liver disease was decreased by 23% after 1000 mg of curcumin intervention. Supplementation with curcumin decreased intestinal cholesterol absorption and further plasma LDL-C levels by inhibiting the intestinal expression of NPC1L1 [186,195]. Curcumin could also elevate the expression of hepatic LDLR via the sterol regulatory element (SRE) pathway [196,197]. However, Tai proved that a 20 μM curcumin treatment enhanced LDL uptake in HepG2 cell by approximately 23%. The mechanism was that curcumin enhanced the density and activity of LDLR through the inhibition of PCSK9 [198]. Tai’s results were supported by another study that curcumin could promote LDLR expression by inhibiting the expression of PCSK9 [199]. Moreover, curcumin was found to decrease PCSK9 expression via reducing the nuclear abundance of HNF-1α, an important PCSK9 regulator [198]. Moreover, curcumin regulated the metabolism of LDL by improving the C-to-U RNA edition of apoB, which is major component of LDL [200]. A total of 0.1% curcumin consumption could also stimulate the conversion of hepatic cholesterol to bile acids via the upregulation of CYP7A1, and by further promoting the removal of LDL (about 56%) [188].

#### 4.2.5. Isoflavone

Genistein, a principal bioactive soy isoflavone, has received great attention due to antioxidative, anti-inflammatory and lipid-lowering effects [201,202]. There exists a considerable amount of evidence supporting the role of genistein in the prevention of cardiovascular disease, osteoporosis, diabetes, and hyperlipemia [203,204,205]. Growing experimental data has showed that genistein consumption made a reduction in LDL-C levels [206,207,208,209]. Genistein supplementation at a dose of 2 g/kg bw significantly reduced the LDL-C level by 40% in high-fat-diet-fed hamsters. In a randomized, double-blind, and placebo-controlled trial, 54 mg of genistein daily for one year were found to decrease the LDL-C level (mean from 108.8 to 78.7 mg/dL) in Caucasian postmenopausal subjects with metabolic syndrome [210]. Similarly, a meta-analysis found that genistein consumption could significantly decrease the LDL-C level in postmenopausal women with metabolic syndrome [211]. Several mechanisms have been proposed to explain the effect of genistein on decreasing the LDL-C level. Genistein could upregulate the expression of hepatic LDLR, thereby inducing the clearance of LDL-C [207]. This result was also verified in vitro studies [212,213]. Moreover, Kartawijaya supported that 40 μM genistein treatment could activate the JNK signaling pathway and SREBP2 processing, which was followed by the upregulation of LDLR [213]. Genistein could also interfere with VLDL assembly. Borradaile found that 50 μM genistein could decrease the secretion of apoB through multiple mechanisms, including inhibiting the expression of HMGR, the activity of ACAT, and the expression and activity of MTP [214].

### 4.3. Stilbenes

Resveratrol is a polyphenolic compound produced by grapes, peanuts, vegetables and other plant species [215]. Polydatin, as the glycoside of resveratrol, is the main bioactive constituent of *Polygonum cuspidatum* Sieb. et Zucc [216]. Studies have demonstrated that resveratrol and its glycoside exhibit a lipid-lowering effect [217,218]. Daily supplementation with 0.02% resveratrol in apoE-deficient mice had been shown to decrease the LDL-C content by 15%. The level of LDL-C was evidently decreased after 3 weeks of treatment with polydatin (25, 50, and 100 mg/kg bw) by 27%, 30%, and 33%, respectively [219]. The underlying mechanisms by which resveratrol and its glycoside mitigate the LDL-C level have been investigated in cell cultures and in animals. Principally, resveratrol and its glycoside increased the expression and activity of hepatic LDLR [220]. Evidence indicated that a high-fat diet plus resveratrol (50 and 100 mg/kg bw) could downregulate the expression of LDLR in rats and further reduce the level of LDL-C [220]. Resveratrol regulated the expression of LDLR via two ways. On the one hand, a 50 μM resveratrol treatment enhanced LDLR transcription via the proteolytic activation of SREBPs and exhibited a 59% increase in the LDL uptake [221]. On the other hand, both 10 μM and 20 μM resveratrol downregulated PCSK9 expression to maintain LDLR levels on the surface of cells [222]. Similarly, 20 μM polydatin upregulated the protein expression level of LDLR and inhibited PCSK9 protein expression, as well as the combination between PCSK9 and LDLR [223]. To a lesser extent, resveratrol also involved bile acid metabolism. Chaothe and Swaan found that resveratrol promoted bile acid transporter ASBT degradation via the ubiquitin–proteasome pathway, which might be associated with an increase in fecal bile acid excretion [224,225]. Shao and colleagues found resveratrol and its liver metabolite resveratrol glucuronide at a dose of 25 μM caused a significant increase in hepatic CYP7A1 and BSEP, indicating the increase in the synthesis and efflux of bile acids [56].

### 4.4. Effect of Other Polyphenols on Lowering LDL-C and Mode of Action

In addition to the above mentioned, polyphenols which exerted LDL-C lowering effects also included protocatechuic acid, vanillic acid, puerarin, kaempferol, and so on. Herein, we focus on the mechanisms by which other dietary polyphenols alleviated the LDL-C level. In vivo study reported that vanillic acid possessed LDL-C lowering potential by inhibiting the HMGR activity [226]. Vanillic acid was orally given (50 mg/kg bw) to hypertensive rats for 4 weeks. The results revealed that vanillic acid reduced the plasma LDL-C level by 64% and inhibited HMGR activity (Table 4). Moreover, an animal study conducted by Ma et al. revealed that the decrease of the plasma LDL-C concentration in the puerarin intervention group was associated with the induction of CYP7A1 [227]. In another study, naringin (25 mg/kg bw) could reduce the plasma LDL-C level in obese mice via inhibiting the expression of SREBP2 and PCSK9 and inducing the expression of LDLR [228]. Kubota and colleagues elucidated the underlying mechanism of lowering LDL-C by ellagic acid [229]. In the presence of 25 μM ellagic acid, the expression of LDLR was significantly increased while extracellular apoB protein and MTP mRNA levels were decreased (*p <* 0.05) (Table 5). Another in vitro study conducted by Ochaiai et al. reported that the uptake of fluorescent-labeled LDL in HepG2 cells was increased after treatment with 100 μM kaempferol for 24 h [230]. The results were attributed to the promotion of LDLR expression and activity in the presence of kaempferol. Moreover, luteolin could affect the cholesterol absorption by regulating the expression of NPC1L1 in Caco-2 cells [136]. Table 4 and Table 5 respectively summarizes the results and details of the listed references based on in vitro or in vivo models.

## 5. Limitations and Suggestions

From the above studies, dietary polyphenols have exhibited the potential effect on lowering the level of LDL-C. However, there are many limitations, which are as follows:

1. In some studies, the crude extracts from food origin were applied to study the LDL-C-reducing activities without determining the active ingredients;
2. In most previous studies, researchers only focused on a partial mechanism about a particular polyphenol without conducting a comprehensive study involved in polyphenol’s potential regulatory pathways;
3. Bioactivity investigations using cell lines have made an extensive use of polyphenols at concentrations in the low-μM-to-mM range. However, after ingestion the dietary polyphenols appear in the circulatory system as phase II metabolites, and their presence in plasma rarely exceeds nM concentrations [12]. There is lack of data which explores the effect of polyphenols metabolites on lowering LDL-C;
4. Researchers paid more attention to in vitro and animal studies but less attention to clinal studies.

Thus, further studies are required to separate and identify the active components in the presence of crude extracts. Moreover, all the potential regulatory pathways about inhibiting the LDL-C level should be adequately considered during research. Furthermore, a comparative study of the effect of polyphenols and their metabolites on lowering LDL-C is needed. Finally, we should focus on clinal studies to provide a necessary dietary reference for people.

## 6. Conclusions

LDL-C-causing CVDs were repeatedly demonstrated in different experimental studies. LDL-C lowering therapies have become a preferred target for CVDs resulting from liner relationship between LDL-C and CVDs risk. LDL-C lowering therapies were mainly involved in hepatic and intestinal target organs. In hepatocytes, the molecular targets contained HMGR, LDLR, PCSK9, IDOL, MTP, apoB, CYP7A1, CETP, SREBPs, squalene synthase, and ACLY, while ABCG5/8, NPC1L1, MTP, ACAT, and ASBT were believed to be associated with the absorption and excretion of cholesterol and bile acid in the gut. From the above studies, dietary polyphenols have exhibited a potential effect on lowering the level of LDL-C via various molecular targets. However, further deep studies are required to elucidate how polyphenols interact with the targets.

## Figures and Tables

**Table 1:** Current strategies for lowering the LDL-C level.

Drug	Mechanism of Action	Adverse Effects	References
Ezetimibe	Inhibition of intestine cholesterol absorption	-	[24]
Mipomersen	ApoB synthesis inhibition	Injection site reactions; Influenza-like symptoms; Fatty liver	[61]
Lomitapide	Reduced secretion of apoB-containing lipoproteins by MTP inhibition	Gastrointestinal tract diarrhea, nausea, vomiting, and dyspepsia; Fatty liver	[70,71]
Statins	Reduced hepatic cholesterol synthesis and increased LDLR expression by blocking hepatic 3-hydroxy-3-methylglutaryl coenzyme A reductase (HMGR)	Statin-associated muscle symptoms (myalgia, myopathy, and myositis with elevated creatinine kinase rhabdomyolysis)	[62,69]
Anti-PCSK9 monoclonal antibodies (mAbs)	Blockade of circulating PCSK9 interaction with LDLR	Injection site reactions	[63]
Bempedoic acid	Reduced hepatic cholesterol synthesis and increased LDLR expression by blocking ATP-citrate lyase (ACLY)	Elevated uric acid levels; Tendon rupture; Increased serum creatinine	[59]
Bile acid sequestrants	Reduced bile acid reabsorption and increased fecal elimination	Gastrointestinal side effects: constipation, bloating, abdominal discomfort, and aggravation of hemorrhoids	[64,72]
CETP inhibitors	Inhibition of CETP	Increase of plasma aldosterone and blood pressure	[60]
Squalene synthase inhibitors	Reduced hepatic cholesterol synthesis and increased LDLR expression by blocking squalene synthase	Acidosis; Elevation of alanine aminotransferase and total bilirubin	[66,73]
ASBT inhibitors	Reduced bile acid reabsorption	Abdominal pain and diarrhea	[67]

**Table 2:** Dietary sources, polyphenols, and cholesterol-lowering activities in animal models.

Dietary Sources	Polyphenols Components	Treatment	Effect	*p*-Value (vs. Model)	References
White tea (*Camellia sinensis*)	-	0.5% aqueous extracts of white tea to diabetes rats for 4 weeks	Decreases in plasma LDL-C	*p <* 0.05	[82]
Green tea (*Camellia sinensis*)	Epigallocatechin gallate, epicatechin, epicatechin gallate, epigallocatechin, gallocatechin gallate, and catechin	0.2% green tea extract was given to atherogenic-diet-fed to SD rats for 4 weeks	Decreases in plasma LDL-C by 40%	*p <* 0.05	[83]
Fuzhuan brick tea (*Camellia sinensis*)	Catechin, epicatechin, epigallocatechin, gallocatechin gallate, epigallocatechin gallate, epicatechin, gallate, rutin, gallic acid, and chlorogenic acid	75 mg/kg, 300 mg Fuzhuan brick tea water extract/kg bw were given to obese rats for 40 days	Reduction in plasma LDL-C by 38%	*p <* 0.05	[84]
Kombucha (*Camellia sinensis*)	-	Kombucha (5 mL/kg bw) was given to hypercholesterolemic-diet-fed rats per day for 16 weeks	Decreases in plasma LDL-C by 36%	*p <* 0.05	[85]
Youcha (*Camellia sinensis*)	-	1500 mg/kg·bw Youcha were respectively given to hyperlipidemia rats for 32 days	Decreases in plasma LDL-C by 24%	*p >* 0.05	[86]
Sanglan tea (*Camellia sinensis*)	15 kinds of flavonoids such as quercetin and kaempferol	Sanglan tea (200 mg/kg bw, respectively) was given to obese mice for 28 weeks	Reduction in plasma LDL-C	*p <* 0.05	[87]
Oil tea (*Camellia sinensis*)	-	4 g/kg bw oil tea was given to type 2 diabetic mice	Lowers plasma LDL-C	*p <* 0.05	[88]
Bowl tea (*Camellia sinensis*)	Gallic acid, epigallocatechin, catechin, L-epicatechin, epigallocatechin gallate, gallocatechin gallate, and epicatechin gallate	Bowl tea (50 mg/kg bw) was given to high-fat-diet-fed mice for 12 weeks	Decreases in plasma LDL-C by 24%	*p <* 0.05	[89]
Persimmon (*Diospyros kaki*) wine and Grape (*Vitis vinifera*) wine	Persimmon wine: epicatechin, epigallocatechin-gallate; Grape wine: catechin, epicatechin, epicatechin-3-O-gallate, and epigallocatechin-gallate	Wine (7.4 ml/kg bw) to atherogenic-diet-fed hamsters for 12 weeks	Decreases in plasma LDL-C levels by 38%	*p <* 0.05	[75]
Yellow wine (*Oryza sativa* L.)	-	Yellow wine polyphenolic compounds (30 mg/kg bw) were given to LDL receptor knockout mice per day for 14 weeks	Decreases in plasma LDL-C by 41%	*p <* 0.05	[90]
Mulberry (*Morus alba* L.)	Gallic acid, protocatechuic acid, 3-caffeoylquinic acid, chlorogenic acid, 4-caffeoyl quinic acid, caffeic acid, rutin, and quercetin-3-O-glucoside	0.5%, 1%, and 2% mulberry water extracts were given to high-fat-diet-fed hamsters for 12 weeks	Lowers plasma LDL-C	*p <* 0.05	[91]
Raspberry (*Rubus idaeus*) juice	Anthocyanins, ellagitannins, and ellagic acid-like compounds	Equivalent to a consumption of 275 mL/day by a 70 kg human to hypercholesterolemic golden Syrian hamsters for 12 weeks	Decreases in plasma LDL-C by 41%	*p <* 0.05	[92]
Bilberry (*Vaccinuim myrtillus*)	Anthocyanins, quercetin-3-O-glucoside, and chlorogenic acid	5 g bilberry powder orally given to Zucker diabetic fatty rats for 8 weeks	Ameliorates LDL-C level by 60%	*p <* 0.05	[8]
Berry (*Lonicera caerulea* L. var. edulis)	Cyanidin-3-glucoside, catechin, and chlorogenic acid	*Lonicera caerulea* berry extract (300 mg/kg bw) was given to high-fat-diet-fed rats for 12 weeks	Decreases in plasma LDL-C by 48%	*p <* 0.05	[93]
White bayberry (*Morella rubra* Sieb. et Zucc.)	Epigallocatechin gallate, epigallocatechin, myricetin-3-O-α-l-rhamnoside, quercetin-3-O-rhamnoside, and kaempferol-3-O-rhamnoside	200 mg/kg bw white bayberry fruit extracts were given to diabetic KK-A^y^ mice for 5 weeks	Ameliorates plasma LDL-C by 58%	*p <* 0.05	[94]
Kiwifruit (*Actinidia deliciosa*)	-	5% of lyophilized kiwifruits were given to atherogenic-diet-fed rats for 33 days	Decreases in plasma LDL-C by 41%	*p <* 0.05	[95]
*Tamarindus indica*	-	500 mg *Tamarindus indica* fruit pulp extract/kg bw to high-cholesterol-diet-fed hamsters for 10 weeks	Lowers plasma LDL-C by 60%	*p <* 0.05	[96]
Apple (*Malus* ssp.)	-	Apple polyphenols (100 mg/kg bw) were given to apolipoprotein-E deficient (ApoE^-/-^) mice for 12 weeks	Decreases in plasma LDL-C	*p <* 0.05	[97]
Apple (*Malus*) juice	Cloudy apple juice: emodin, kaempferol, cyanidin cation, stevioside, and butylated hydroxytoluene	Cloudy apple juice (15 ml/kg bw) was given to diabetic rats for 21 days	Decreases in plasma LDL-C by 74%	*p <* 0.05	[98]
Hawthorn (*Crataegus oxyacantha*)	Chlorogenic acid, epicatechin, rutin, and hyperoside	400 mg/kg bw extracts from hawthorn fruit peel and flesh were respectively given to high-fructose-diet-fed mice for 8 weeks	Reduction in plasma LDL-C by 39%	*p <* 0.05	[99]
Ajwa date (*Phoenix dactylifera* L.)	-	Ajwa date extract (25 mg/kg bw) was given to hypercholestrolemic rats for 28 days	Ameliorates plasma LDL-C	*p <* 0.05	[100]
*Hibiscus sabdariffa*	18 phenolic compounds	100 mg/kg bw *Hibiscus sabdariffa* polyphenolic extract to diabetic rats for 7 weeks	Reduction in plasma LDL/HDL ratio	*p <* 0.05	[101]
*Hibiscus sabdariffa*	Protocatechuic acid, catechin, gallocatechins, caffeic acid, and gallocatechin gallates	0.5% *Hibiscus sabdariffa* extract were given to obese hamsters for 10 weeks	Ameliorates plasma LDL-C	*p <* 0.05	[102]
Propolis	Green propolis: artepellin c, pinocembrin, and kampferol; Red propolis: 3-Hydroxy-8,9-dimethoxypterocarpan, medicarpin, and daidzein; Brown propolis: pinocembrin, caffeic acid phenyl ester, quercetin, and galangin	Green, red, or brown propolis extract (250 mg/kg bw) to cholesterol-enriched-diet-fed LDLR knockout mice for 4 weeks	Reduces plasma non-HDL-C treated with the red propolis by 17%	*p <* 0.05	[103]
*Aloe vera*	-	1.25 g *Aloe vera* extracts/kg bw was given to type 2 diabetic rats for 28 days	Lowers plasma LDL-C	*p >* 0.05	[104]
Adlay (*Coix lachryma-jobi* L. var. *ma-yuen* Stapf)	Gallic acid and catechin	40 mg total phenolics/kg bw to high-cholesterol-diet-fed rats for 28 days	Decreases in plasma LDL-C by 30%	*p <* 0.05	[105]
Bergamot (*Citrus bergamia* Risso et Poiteau)	-	50 mg/kg bw bergamot polyphenolic formulation was given to high-fat-diet-fed mice for 16 weeks	Reduction in plasma LDL-C by 40%	*p <* 0.05	[106]
*Perilla. frutescens* (L.) Britt.	-	Leaf extracts (250 mg/kg bw) were given to high-fat-diet-fed rats for 4 weeks	Lowers plasma LDL-C by 53%	*p <* 0.05	[78]
Onion (*Allium cepa* L.)	Quercetin and isoquercitrin	Hyperlipidemic rats treated with onion extract at 4.5g/kg bw for 4 weeks	Ameliorates plasma LDL-C by 45%	*p <* 0.05	[79]
*Rhodomyrtus tomentosa* fruit juice	-	2 g/kg bw frozen fruit juice was given to high-fat-diet-fed rats for 75 days	Reduces plasma LDL-C	*p <* 0.05	[107]
*Astragalus radix*	-	25 mg *Astragalus radix* total flavones/kg bw was given to diabetic mice for 8 weeks	Lowers plasma LDL-C	*p <* 0.05	[108]
Turmeric (*Curcuma longa*)	-	2.0% turmeric powder was given to high-fat-diet-fed mice for 8 weeks	Lowers plasma LDL-C	*p <* 0.05	[109]
*Schinus terebinthifolius Raddi*	Gallic acid, catechin, naringenin, and kaempferol	50 mg *Schinus terebinthifolius Raddi* extract/kg bw to high-cholesterol-diet-fed rats for 9 weeks	Ameliorates plasma LDL-C by 41%	*p <* 0.05	[110]

**Table 3:** Dietary sources, polyphenols, and cholesterol-lowering activities in clinical studies.

Dietary Sources	Polyphenols Components	Treatment	Effect	*p*-Value	References
Green tea (*Camellia sinesis*)	Epigallocatechin gallate, epicatechin, epigallocatechin, epicatechin gallate, and gallocatechin gallate	Green tea capsules were given to healthy postmenopausal women for 2 months	Reduction in plasma LDL-C by 8% from baseline	*p <* 0.05 (vs. placebo group)	[76]
Green tea (*Camellia sinesis*)	Epigallocatechin gallate, epicatechin gallate, epigallocatechin, epicatechin, and gallocatechin gallate	1500 mg green tea extract was given to women with central obesity for 12 weeks	Decreases in plasma LDL-C by 10% from baseline	*p <* 0.05 (vs. baseline)	[111]
Green tea (*Camellia sinesis*)	Epigallocatechin 3-gallate, epigallocatechin, catechin, epicatechin, gallocatechin gallate, and epicatechin-3-gallate	1500 mg STA-2 (a pharmaceutical preparation of green tea polyphenols) daily was given to patients with chronic stable angina for 6 weeks	Decreases in plasma LDL-C	*p <* 0.05 (vs. placebo group)	[112]
Green tea (*Camellia sinesis*)	Catechins and epigallocatechin-3-gallate	400 mg of decaffeinated green tea extract daily was given to patients with type 2 diabetes mellitu for 12 weeks	Decreases in plasma LDL-C by 9% from baseline	*p >* 0.05 (vs. placebo group)	[113]
Goishi tea (*Camellia sinesis*)	-	195 ml Goishi tea drink was given to hypercholesterolaemia individuals for 12 weeks	No changes in plasma LDL-C	*p >* 0.05 (vs. placebo group)	[114]
Red wine (*Vitis vinifera* L.)	Resveratrol	2 capsules daily nonalcoholic red wine extract were given to nondiabetic humans for 8 weeks	Decreases in plasma LDL-C by 4% from baseline	*p <* 0.05 (vs. baseline)	[115]
Chokeberry (*Aronia melanocarpa* L.) juice	Cyanidin 3-galactoside, cyanidin 3-arabinoside, and cyanidin 3-xyloside	200 mL chokeberry juice given to hypertensive subjects per day for 4 weeks	Reduction in plasma LDL-C by 7% from baseline	*p >* 0.05 (vs. baseline)	[9]
Aronia berry (*Aronia melanocarpa*)	Anthocyanins, hydroxycinnamic acids, and proanthocyanidins	500 mg aronia extract per day was given to healthy adults for 12 weeks	Reduction in plasma LDL-C by 11% from baseline	*p <* 0.05 (vs. placebo group)	[116]
Blueberry (*Vaccinium virgatum*.)	-	22 g freeze-dried blueberries daily were given to men with type 2 diabetes for 8 weeks	Decreases in plasma LDL-C by 25% from baseline	*p <* 0.05 (vs. placebo group)	[117]
Grape (*Vitis vinifera* L.)	Myricetin, quercetin, catechin, epicatechin, epigallocatechin, catechin gallate, and ellagic acid	700 mg grape extract was given to healthy volunteers for 56 days	Reduces plasma LDL-C by 15% from baseline	*p <* 0.05 (vs. placebo group)	[118]
Grape (*Vitis vinifera* L.)	-	500 g Condori red grapes daily were given to hypercholesterolemic humans for 8 weeks	Reduces plasma LDL-C with grapes treatment by 15%	*p <* 0.01 (vs. baseline)	[119]
Grapefruit (*Vitis vinifera* L.), bitter orange (*Citrus aurantium* Linné), and olive (*Olea europaea*)	Naringin, narirutin, rhoifolin, poncirin, apigenin, neohesperidin, neodiosmin, luteolin, and oleuropein	1000 mg grapefruit, bitter orange, and olive leaf extracts were given to healthy subjects for 8 weeks	Reduces plasma LDL-C by 9% from baseline	*p <* 0.05 (vs. placebo group)	[120]
Grape (*Vitis vinifera* L.)	-	Overweight individuals were assigned to receive grape seed extract (300 mg/day) for 12 weeks	Reduces plasma LDL-C by 16% from baseline	*p <* 0.05 (vs. placebo group)	[80]
Olive (*Olea europaea*)	-	250 mg/day olive extract was given to postmenopausal women for 12 months	Decreases in plasma LDL-C by 21% from baseline	*p <* 0.05 (vs. placebo group)	[121]
Olive (*Olea europaea*)	-	400 g yogurt with 50 mg of encapsulated olive polyphenols was given to volunteers for 2 weeks	Ameliorates plasma LDL-C by 6% from baseline	*p <* 0.05 (vs. baseline)	[122]
Pomegranate (*Punica granatum* L.)	Ellagic acid	1000 mg pomegranate extract was given to obese individuals for 30 days	Reduction in plasma LDL-C by 10% from baseline	*p <* 0.01 (vs. baseline)	[123]
Pomegranate (*Punica granatum* L.) juice	-	200 mL/day pomegranate juice was given to type 2 diabetes patients for 6 weeks	Reduces plasma LDL-C by 10% from baseline	*p <* 0.05 (vs. baseline)	[124]
Cherry (*Prunus avium*) juice	-	480 mL cherry juice drink daily to older adults for 12 weeks	Decreases in plasma LDL-C by 3% from baseline	*p <* 0.05 (vs. placebo group)	[125]
Tart cherry (*Prunus cerasus*)	Cyanidin sophoroside, cyanidin glucosylrutinoside, cyanidin-glucoside, cyanidin xylosylrutinoside, cyanidin rutinoside, and peonidin rutinoside	240 mL of tart cherry juice twice daily was given to adults with metabolic syndrome for 12 weeks	Reduction in plasma LDL-C by 15% from baseline	*p >* 0.05 (vs. baseline)	[77]
*Ecklonia cava*	Dieckol, 8,8′-bieckol, 6,6′-bieckol, and phlorofurofucoeckol A	72 mg *Ecklonia cava* polyphenols per day to overweight Korean individuals for 12 weeks	Ameliorates plasma LDL-C by 10% from baseline	*p <* 0.05 (vs. placebo group)	[126]

*p* < 0.05 considered to be statistically significant.

**Table 4:** Effect of other polyphenols on LDL-C and their mechanism based on in vivo models.

Subclasses	Polyphenol	Study Type	Results	Mechanism	References
Phenolic acid	Protocatechuic acid	0.05% (*w*/*w*) given to cholesterol-fed rats for 4 weeks	Lowers levels of non-HDL-C from 0.88 ± 014 mmol/L to 0.74 ± 0.04 mmol/L (*p <* 0.05)	Increases the expression of hepatic LDLR	[231]
Phenolic acid	Vanillic acid	Vanillic acid (50 mg/kg bw) to hypertensive rats for 4 weeks	Decreases in plasma LDL-C by 64% (*p <* 0.05)	Inhibits HMGR activity	[226]
Isoflavone	Puerarin glycosides	Puerarin glycosides (0.1%) was given to mice for 3 weeks	Reduction in plasma TC levels (*p <* 0.05)	Increases the expression of LDLR; Downregulates the transcription and translation of HMGR	[232]
Isoflavone	Puerarin	Orally (200 mg/kg bw and 400 mg/kg bw) puerarin administered to hyperlipidaemia mice for 8 weeks	Decreases in plasma LDL-C level (*p <* 0.05)	Regulates the expression of phosphorylated JNK, phosphorylated c-Jun protein, and CYP7A1	[227]
Flavones	Apigenin	Apigenin was orally administrated to high-fat-fed mice	Decreases the level of LDL-C in plasma by 19%, 16%, and 55%, respectively (*p <* 0.05)	Promotes the absorption of hepatic LDL-C and increases the transformation of hepatic cholesterol into bile acid by regulating LDLR and CYP7A1 expression	[233]
Flavones	Apigenin	Apigenin (60 ppm and 300 ppm) given orally to hamsters with hypercholesterolaemia	Reduces the nonHDL-C level by 40% and 41% (*p <* 0.05)	Reduces the uptake of dietary cholesterol by inhibiting NPC1L1; Stimulates hepatic LDLR expression	[234]
Flavones	Luteolin	Mice were administered daily 50 mg/kg bw of luteolin in addition to ethanol exposure	Reduces LDL-C level in plasma by 52% (*p <* 0.05)	Inhibits cholesterol biosynthesis by regulating SREBP2 and HMGR	[235]
Flavones	Luteolin	Luteolin (1.5%) was given to high-fat-fed mice for 57 days	Reduces LDL-C levels by 33% (*p <* 0.05)	Suppresses HNF4α targeted genes, such as MTP, apoB	[236]
Flavones	Luteolin	Luteolin (50 mg/kg bw) given orally to hyperlipidemia rats for 6 weeks	Reduces LDL-C levels by 34% (*p <* 0.05)	Increases CYP7A1 activities in liver	[81]
Flavones	Luteolin-7-glucoside	Luteolin-7-glucoside (2 mg/kg bw) orally to rats	Decreases levels of LDL-C in plasma by 40% (*p <* 0.05)	Decreases cholesterol synthesis via decreasing HMGR expression	[237]
Flavanones	Naringin	Naringin (25 mg/kg bw) given orally to obese mice for 8 weeks	Decreases the level of LDL-C in plasma (*p <* 0.05)	Downregulates the expression of SREBP2 and PCSK9, and upregulates the expression of LDLR through AMPK activation	[228]
Ligans	Leoligin	0.14 mg leoligin was given to CETP transgenic mice for 7 days	Reduces LDL-C level in plasma	Activates CETP activity	[238]
Ligans	Sesamin	Hamsters were fed two experimental diets containing 0.02% sesamin or 0.5% sesamin for 6 weeks	Lowers plasma non-HDL-C level by 25% and 32% (*p <* 0.05)	Reduces cholesterol absorption by inhibiting intestinal NPC1L1, ACAT2, MTP, and ABCG5/8; Stimulates LDLR and CYP7A1 expression	[239]

**Table 5:** Effect of other polyphenols on LDL-C and their mechanism based on in vitro models.

Subclasses	Polyphenol	Study Type	Results	Mechanism	References
Phenolic acid	Ellagic acid	25 μM ellagic acid to HepG2 cells	Regulates cholesterol metabolism	Upregulates of LDLR, downregulates MTP mRNA and extracellular apoB levels	[229]
Flavonols	Kaempferol	The HepG2 cells were incubated for 24 h with kaempferol (100 μM)	Increases fluorescent-labeled LDL uptake (*p <* 0.05)	Stimulates the expression of LDLR through activating LDLR transcription factor Sp1	[230]
Flavones	Luteolin	Caco-2 cells were incubated with 100 μM luteolin	Inhibits cholesterol uptake in Caco-2 cells (*p <* 0.05)	Inhibits intestinal cholesterol absorption mediated by NPC1L1	[136]
Flavones	Rutin	Caco-2 cells were treated with 115.7 μM rutin; Rutin (17.85 μM) was used to measure HMGR activity inhibition	Lowers the amount of cholesterol in the intracellular compartment; Reduces HMGR activity by 50% (*p <* 0.05)	Inhibits the uptake of dietary cholesterol and the activity of HMGR	[240]
Flavanones	Hesperetin	HepG2 cells were exposed to hesperetin (100 μM)	Induces the activity of LDLR promoter (*p <* 0.05)	Induces the transcription of LDLR through SREBPs	[241]
Flavanones	Glucosyl hesperidin	HepG2 cells were treated with 0.8 mM and 1.2 mM glucosyl hesperidin	Reduces cellular cholesteryl ester content (*p <* 0.05)	Suppresses the secretion of apoB	[242]
